# A new interdisciplinary treatment strategy versus usual medical care for the treatment of subacromial impingement syndrome: a randomized controlled trial

**DOI:** 10.1186/1471-2474-8-15

**Published:** 2007-02-22

**Authors:** Oscar Dorrestijn, Martin Stevens, Ron L Diercks, Klaas van der Meer, Jan C Winters

**Affiliations:** 1 Department of Orthopedic Surgery, University Medical Center Groningen, University of Groningen, Hanzeplein 1, 9713 GZ Groningen, The Netherlands; 2 Department of General Practice, University Medical Center Groningen, University of Groningen, Antonius Deusinglaan 1, 9713 AV Groningen, The Netherlands

## Abstract

**Background:**

Subacromial impingement syndrome (SIS) is the most frequently recorded shoulder disorder. When conservative treatment of SIS fails, a subacromial decompression is warranted. However, the best moment of referral for surgery is not well defined. Both early and late referrals have disadvantages – unnecessary operations and smaller improvements in shoulder function, respectively. This paper describes the design of a new interdisciplinary treatment strategy for SIS (TRANSIT), which comprises rules to treat SIS in primary care and a well-defined moment of referral for surgery.

**Methods/Design:**

The effectiveness of an arthroscopic subacromial decompression versus usual medical care will be evaluated in a randomized controlled trial (RCT). Patients are eligible for inclusion when experiencing a recurrence of SIS within one year after a first episode of SIS which was successfully treated with a subacromial corticosteroid injection. After inclusion they will receive injection treatment again by their general practitioner. When, after this treatment, there is a second recurrence within a year post-injection, the participants will be randomized to either an arthroscopic subacromial decompression (intervention group) or continuation of usual medical care (control group). The latter will be performed by a general practitioner according to the Dutch National Guidelines for Shoulder Problems. At inclusion, at randomization and three, six and 12 months post-randomization an outcome assessment will take place. The primary outcome measure is the patient-reported Shoulder Disability Questionnaire. The secondary outcome measures include both disease-specific and generic measures, and an economic evaluation. Treatment effects will be compared for all measurement points by using a GLM repeated measures analyses.

**Discussion:**

The rationale and design of an RCT comparing arthroscopic subacromial decompression with usual medical care for subacromial impingement syndrome are presented. The results of this study will improve insight into the best moment of referral for surgery for SIS.

## Background

Shoulder disorders are encountered frequently in general practice. In a Dutch study the cumulative incidence of shoulder problems was estimated to be 23.1/1000 patients/year [1]. For the neck and upper extremity it was the most commonly presented musculoskeletal complaint. A differentiation between various diagnoses of shoulder problems in general practice was presented in another Dutch study [2]. Subacromial impingement syndrome (SIS) was the most frequently recorded disorder (44%).

The primary treatment of SIS is conservative. In primary health care a broad spectrum of conservative treatments for SIS is available: rest, non-steroidal anti-inflammatory drugs (NSAIDs), corticosteroid injections, physiotherapy and manual therapy. In the Netherlands, the choice of treatments for shoulder conditions is proposed by the National Guidelines for Shoulder Problems, published by the Dutch College of General Practitioners [3]. If patients do not respond sufficiently to these nonoperative measures, referral to an orthopedic surgeon for evaluation for (arthroscopic) subacromial decompression is recommended [4]. The best moment of referral is not well defined though, so a therapeutic dilemma for the general practitioner exists: how many different treatments from the spectrum of nonoperative interventions should be repeated or tried out if previous ones have failed? And how long should one wait for recovery before referring? The preoperative duration of symptoms reported in different articles published in the last two decades on surgery for SIS is quite long, ranging from an average of 18 to 40 months [5-9]. Expert opinions advocate orthopedic referral is warranted for patients who do not respond to nonoperative measures after (three to) six months [10-13]. Moreover, several observational studies report a significantly better outcome of surgery in patients who had a short symptom duration compared to those who had prolonged symptoms before surgery [6,9]. From these studies it seems that the moment of referral is crucial. However, approximately 60% of the patients recover within 27 months with nonoperative measures, which has to be taken into consideration [14]. Early surgery would therefore not always be appropriate because patients could recover nonoperatively. On the other hand, late surgery might lead to smaller improvements of shoulder function. To improve insight, we designed an interdisciplinary treatment strategy called TRANSIT (TRANSmural treatment strategy for Subacromial ImpingemenT), which contains rules to treat patients with SIS in primary care and a well-defined moment of referral to an orthopedic surgeon for arthroscopic acromioplasty. The TRANSIT outline for the treatment of SIS will be tested in a randomized controlled trial (RCT), comparing treatment results of participants allocated to arthroscopic subacromial decompression with continuation of usual medical care by the general practitioner. The present paper reports on the content of TRANSIT and the methodological design of this RCT.

## Methods/Design

### Study design

**Figure 1:**
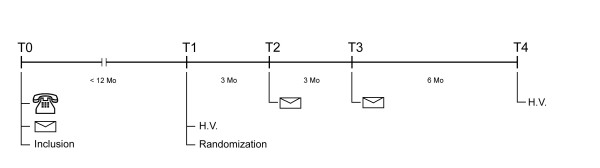
**Study design and follow-up procedures**. **Follow-up procedures**. At T0, potential participants are contacted by phone. After informed consent is received, patients are included. At T0, T2 and T3 questionnaires will be returned by mail. At T1 and T4 participants visit the hospital (H.V.).

The study is designed as a randomized controlled trial to evaluate the effectiveness of a new interdisciplinary treatment strategy for SIS. Figure 1 presents the design. The Medical Ethics Committee of University Medical Center Groningen has approved the study design, the protocols and the informed consent procedure. Participants are assigned at random to the control or to the intervention group. The follow up period after randomization is 12 months.

### TRANSIT: an interdisciplinary strategy

Initially, TRANSIT follows the National Guidelines for Shoulder Problems to diagnose and treat patients with SIS [3]. For all new patients, treatment starts with NSAIDs. If results following a maximum of two weeks of treatment are insufficient, therapy is continued with a subacromial corticosteroid injection. When ineffective, this injection is repeated within one month. In case of recurrence within 12 months after the first successful subacromial injection, eligible patients (see "selection of participants") are asked to participate in the study. Recurrence means patients having pain again, having increased pain or no longer experiencing pain relief. Included patients will receive another subacromial injection from their general practitioner, which if necessary can be repeated within one month. In case of a second recurrence within 12 months after the last successful injection, participants will be randomized to either an arthroscopic subacromial decompression performed within four weeks or continuation of treatment by the general practitioner according to the National Guidelines for Shoulder Problems. Participants who have a recurrence more than 12 months after the last injection will not be randomized. Patients who do not respond to two injections within one month will not be included either. Their long preoperative duration does not fit within the concept of this study, in which participants have surgery after an on average shorter-than-usual duration of symptoms.

For both NSAIDs and subacromial corticosteroid injections there is evidence of their effectiveness for SIS, albeit for the short term (up to a nine-month period) [15,16]. The reason for repeating a subacromial injection within a month in case of ineffectiveness is to target inaccuracy of subacromial injections. Several studies have reported on accuracy rates, ranging from 60 to 80% [17-20]. In addition to being a treatment, the subacromial injection constitutes a diagnosis itself (Neer impingement test) [13]. The injection fluid is a mixture of a corticosteroid and a local anesthetic (lidocaine). When a subacromial injection eliminates the pain immediately (as a result of the injected lidocaine), it confirms the diagnosis of SIS. If the injection does not eliminate the pain immediately, the diagnosis might be wrong or the injection could have been placed inaccurately. Furthermore, a positive reaction on a subacromial injection predicts better patient recovery following arthroscopic subacromial decompression compared to patients who have a negative reaction but a confirmed diagnosis through imaging [21].

### Setting

The trial is carried out in 50 general practices within an area of 20 kilometers from University Medical Center Groningen (UMCG) and at the Department of Orthopedic Surgery of UMCG. A total of 160 general practices in the surrounding area of Groningen were sent letters inviting them to participate in this study. Forty interested general practitioners attended an information meeting about the study protocol. Following a standard protocol, they were instructed in injecting into the subacromial space, which they subsequently practiced on phantoms and fresh-frozen cadaver shoulders. Another 10 general practitioners who could not attend the meeting were visited at their practice to be informed about the trial.

### Study population

#### Sample size

The aim is to include 70 participants in the study. This number is based on the assumption that one year after randomization 50% of the participants will have recovered with conservative treatment [22,23] and 85% will successfully recover by means of arthroscopic subacromial decompression. The latter assumption is based on recovery rates presented in earlier studies in which successful results of arthroscopic subacromial decompression are reported in 86 to 95% of the cases [24-26]. A power analysis has been based on the effects of these treatments on shoulder function. In this study shoulder function will be measured with the Shoulder Disability Questionnaire, which is an outcome measure comparable to the instruments used in the referred articles. In order to detect a clinically relevant difference in shoulder function (35% differences in means) one year after randomization between the intervention and control group, 64 participants are needed – 32 in each group. These numbers are based on a power (1-B) of 0.80 and a significance level of 5% (two-sided). When a dropout rate of 10% is taken into account, 70 participants are to be included.

#### Selection of participants

Subjects participating in the study are recruited by the general practitioners involved. They introduce the study to patients who seem eligible and give interested patients a brochure about the trial, to be read at home. After having received consent, the general practitioners fax the name and telephone numbers of the interested patients to the research team. Subsequently the researcher calls the interested patients within one week. During this conversation the aim and implications of the study are explained again and the eligibility criteria are checked. Patients are eligible for participation when they meet the inclusion and exclusion criteria presented in Table 1.

If patients meet these criteria and wish to join the study, an informed consent form is signed before participation begins. The new participants receive another subacromial corticosteroid injection from the general practitioner for treatment of their first recurrence. If ineffective, this injection will be repeated within one month. Participants who have a recurrence of problems within one year after the last injection will contact their general practitioner, who will inform the researcher by fax. Subsequently the participant will be invited to visit the researcher at UMCG for a physical outcome measurement and to be randomized to one of the two treatment groups. Participants who do not have a recurrence within one year will not be randomized.

### Randomization

Participants will be block-randomized into two groups: surgery or usual medical care. Subsets of four participants are made per participating general practitioner. Two participants will be assigned to the treatment group and two to the control group. This process is repeated as the trial progresses. Block randomization is a method used to prevent unequal treatment-group sizes [27]. In this study, this method is used to ensure more or less equal treatment groups per general practitioner. It prevents participants referred by one single general practitioner from being all treated according to usual medical care or surgery.

Sealed, opaque envelopes in subsets of four per general practitioner are used for randomization. The envelopes look identical and have identification for the referring general practitioner as well as a sequential number for the subset. A random sequence of envelopes is generated by an independent person. The participants choose one envelope under supervision of the researcher.

### Interventions

The treatments the two study groups are assigned to are not different from those in usual medical care. The only difference is that the surgery group, in most cases, will have an operation after a shorter preoperative duration of symptoms compared to patients who fail to respond to conservative measures in usual medical care.

The operative treatment is an arthroscopic subacromial decompression performed within four weeks after randomization. Preoperatively no imaging will be performed, except for a shoulder radiograph. This is because the positive reactions to the previous injections have confirmed the SIS diagnosis. The operation is carried out in day surgery under general anesthesia, possibly extended with a regional nerve block for postoperative pain reduction. During the operation the patient is in a beach-chair position. The arthroscopy starts with an inspection of the glenohumeral joint, the intra-articular surface of the rotator cuff and the biceps tendon. Then the endoscope is introduced in the subacromial bursa. Subsequently the treatment consists of a bursectomy with partial resection of the anteroinferior part of the acromion and the coracoacromial ligament. If seen, tears of the rotator cuff will be noted but not repaired – the reason being that there is little evidence to either support or refute the efficacy of common interventions for rotator cuff tears [28]. Therefore, an ongoing discussion exists as to whether to operate on tears of the rotator cuff. As most rotator cuff tears are caused by degeneration, which is confirmed by histochemical and morphometrical research [29], an operation consisting of suturing degenerated tissue is not expected to be effective in the long term because of the ongoing process of postoperative degeneration and the associated risk of retears [30,31]. In this study, all participants have a painful arc syndrome and a positive impingement test. These patients can have a partial thickness rotator cuff tear, or in the worst case a small full-thickness rotator cuff tear. Any patients with a history and clinical symptoms of a massive rotator cuff tear (i.e. an inability to reach overhead, lift with an outstretched arm, and an impairment of pushing and pulling) will be excluded. As the outcome measures of this study focus on pain and functioning of the shoulder and not on the integrity of the rotator cuff, the extent of the damage, on the continuum from no tear to a small full-thickness tear, has no consequences for the study groups.

One senior surgeon (RLD) will undertake all procedures. Before discharge the participant receives a sling and instructions for daily pendulum exercises. Two weeks post-surgery the participant visits the clinic for wound inspection. New instructions will follow for home training exercises which focus on increasing the range of motion of the shoulder. Four weeks later the participant may start exercises for strengthening the rotator cuff muscles. If indicated, physiotherapy can be part of the rehabilitation process.

The group randomized to continuation of usual medical care will receive treatment prescribed by the general practitioner according to the Guidelines for Shoulder Problems of the Dutch College of General Practitioners [3]. In primary health care a broad spectrum of conservative treatments for subacromial impingement syndrome is available: rest, nonsteroidal anti-inflammatory drugs, corticosteroid injections, physiotherapy and manual therapy. If needed, the general practitioner can also refer the participant to a hospital of random choice for further assessment and/or to be evaluated for surgery.

### Outcome assessment

At inclusion (T_0_), at randomization (T_1_) and at three (T_2_), six (T_3_) and 12 months (T_4_) post-randomization, outcome assessment will take place in both study groups (Table 2). At all measurement points, outcome will be assessed by means of questionnaires which are sent to the participants by mail three days earlier. At T_1 _and T_4 _the participants are asked to visit the researcher at UMCG for an additional physical assessment. The questionnaires addressing those measurement moments can be filled in at home and be handed in at the patient's visit. The researcher checks all questionnaires for missing or incorrect data.

The outcome measures used focus on shoulder function, pain and health-related quality of life. They are disease-specific or generic, and from a patient- or physician-based perspective. The following applied measures are disease-specific and patient-based: the Shoulder Disability Questionnaire, the Shoulder Pain Score and the Shoulder Rating Questionnaire. The Individual Relative Constant Score is a disease-specific as well as a patient- and physician-based instrument. The Short-form 36 Health Survey and the Patient-perceived recovery are both generic and patient-based. For the cost effectiveness analysis a generic, patient-based questionnaire will be used. The specific characteristics of the outcome measures will be mentioned below.

#### Primary outcome measure

##### Shoulder Disability Questionnaire (SDQ)

The SDQ is a 16-item measure for functional status limitation in patients with shoulder disorders and assesses the past 24 hours [32]. The 16 questions can be answered with either yes, no or not applicable. The score is calculated by multiplying the yes/no ratio by 100.

#### Secondary outcome measures

##### The individual relative constant score

This shoulder assessment score is a modification of the Constant-Murley shoulder score, in which patient-reported subjective assessment and objective measurement of shoulder function takes place at a ratio of35:65 [33]. The system is divided into subjective measures for pain and daily activities and objective measures for range of motion (max. 75 points) and power (max. 25 points). The modified score contains the same items as the original score, but uses the functional performances of the uninjured collateral shoulder of the same individual as a reference [34]. It is expected to be more reliable for larger and incoherent patient populations because specific interindividual differences regarding the patient's age, gender and constitution as well as other individual physiological parameters are eliminated.

Shoulder strength measurement, which is a part of the Constant score, is performed according to a standard method, as proposed by Bankes et al. [35]. A digital dynamometer, the Handyscale^®^, is used and validated for this application [36]. It measures a maximum of 15 kilograms with two decimals and an interval of 20 grams. The test position is the subject standing with the arm in 90° elevation in the scapular plane, elbow extended and forearm pronated. An adjustable strap is placed around the forearm just proximal to the radiocarpal joint and attached to the Handyscale^®^. The dynamometer is firmly attached to a solid surface. The subjects are instructed to pull upward with maximum effort until requested to stop. The reading of the dynamometer is taken after five seconds of maximum effort. For both the uninjured and the affected arm, three successive maximum pulls will be obtained. The highest value out of these three provides the strength score for each arm. Patients unable to reach the test position will receive the value of zero. The scores for strength assessment in the Constant-Murley score range from zero to 25 pounds, hence to calculate the individual relative strength score the ratio of the maximum strength scores of the affected and the unaffected arm is multiplied by 25.

The scores for the other individual parameters range from zero to 75 points. To calculate the individual relative sum score for these items, the ratio of these scores for the affected and the unaffected arm will be multiplied by 75. The individual relative Constant score is calculated by adding the individual relative strength score and the individual relative sum score. The maximum attainable score is 100 points.

##### The Shoulder Pain Score (SPS)

The SPS is a questionnaire to assess pain experienced by patients with shoulder disorders and includes a 24-hour recall frame [37]. The score consists of six pain symptom questions and a 101-Numerical Rating Scale (NRS-101). The SPS has been proved to be a useful instrument for following the course of the disorder over time, and gives an indication when a patient feels cured. Each question receives a maximum of four points. The NRS-101 is also transposed to a four-point scale (0–9 = 1, 10–39 = 2, 40–69 = 3 and 70–100 = 4). The minimum SPS score is seven points, the maximum score 28.

##### Shoulder Rating Questionnaire (SRQ)

The SRQ is a self-administered patient-based instrument which assesses shoulder function in 19 multiple-choice questions covering seven domains [38,39]. Five subscales are graded separately by averaging the scores of the completed questions, multiplied by two and a weighting factor. The SRQ comprises two additional dimensions compared to the SDQ: recreational and athletic activities and work. The sum scores range from minimum 17 to maximum 100 points.

##### Short-form 36 Health Survey (SF-36)

The SF-36 is a generic health status measure. It is composed of 36 questions and standardized response choices, organized into eight multi-item scales: physical functioning, role limitations due to physical health problems, bodily pain, general health perceptions, vitality, social functioning, role limitations due to emotional problems, and general mental health [40,41]. For purposes of this study we used the standard version of the questionnaire, covering a four-week time frame. All raw scale scores are linearly converted to a zero-to-100 scale, with higher scores indicating higher levels of functioning or well-being.

##### Patient-perceived recovery (PPR)

In addition to the SF-36 there is the PPR, a one-item score concerning recovery following treatment, measured on a seven-point ordinal scale [42].

##### Cost effectiveness

An economic evaluation will be performed using a questionnaire to assess direct health care costs as well as direct non-health related costs. The questionnaire is composed of 24 questions regarding costs of the last six months. The data will be used for a cost-effectiveness analysis, which will be done by the UMCG Medical Technology Assessment office.

### Statistical analyses

To estimate the effect of the interventions, analyses will be performed using SPSS 12.0 for the outcome measures. The baseline characteristics from both study groups will be compared for equality by means of an Independent Samples T-test (p-value 0.05) for continuous variables and a chi-square test for dichotomous variables. To compare treatment effects from measurement points T_0 _to T_4_, a GLM repeated measures analyses will be performed. Data will be analyzed according to the intention-to-treat principle and the per-protocol principle.

## Discussion

TRANSIT is designed to test if early referral for surgery leads to earlier and more complete improvement in shoulder pain and function than continuation of usual medical care for patients suffering from SIS. This has been advocated in expert opinions, but has never been proven in a randomized controlled trial.

The results of this study will improve insight into the best moment of referral for surgery for SIS. If, in the TRANSIT outline, participants who have had an arthroscopic subacromial decompression prove to have better results than those who continued with usual medical care, a future update of Dutch and/or international guidelines for shoulder conditions will be needed.

The rationale and design of an RCT comparing a new interdisciplinary treatment strategy with usual medical care for subacromial impingement syndrome have been presented.

## Competing interests

The author(s) declare that they have no competing interests.

## Authors' contributions

JCW, MS and RLD originated the idea for the study. JCW, MS, OD and RLD contributed to its design and developed the intervention protocol. OD is responsible for the data collection and drafted the manuscript. All authors (JCW, KvdM, MS, OD and RLD) have read and corrected draft versions of the manuscript and approved the final manuscript.

## Pre-publication history

The pre-publication history for this paper can be accessed here:



## Figures and Tables

**Table 1:** Patient eligibility criteria

**Inclusion criteria:**
1. Pain upon abduction of the shoulder with painful arch;
2. Shoulder pain as a recurrence of an episode with a maximum duration of 12 months in which a partial or good response is achieved with subacromial corticosteroid injection(s);
3. A maximum duration of six months of shoulder problems prior to the first subacromial injection, possibly treated with non-steroidal anti-inflammatory drugs (NSAIDs) and/or physiotherapy;
4. No shoulder problems for at least two years prior to the current episode of shoulder pain;
5. Men and women, aged between 30 and 60 years;
6. Being able to give an informed consent.

**Exclusion criteria:**

1. Shoulder girdle pain;
2. Shoulder pain not based on pain upon abduction of the shoulder;
3. Signs of cervical root compression;
4. Bilateral shoulder pain;
5. Secondary subacromial impingement;
6. Presence of specific rheumatic diseases;
7. History of severe trauma of the shoulder within the previous two years (e.g. fracture, luxation);
8. History and/or clinical symptoms of a large rotator cuff tear;
9. Previous surgery of the affected shoulder;
10. Extrinsic causes of shoulder pain;
11. Presence of dementia or other psychiatric disorders;
12. Not being able to fill in questionnaires in Dutch.

**Table 2:** Follow-up measurements

**Outcome measures**	**T_0_**	**T_1_**	**T_2_**	**T_3_**	**T_4_**
SDQ	+	+	+	+	+
SPS	+	+	+	+	+
SRQ	+	+	+	+	+
PPR		+	+	+	+
SF-36		+			+
Cost effectiveness		+		+	+
IRCS		+			+

Abbreviations used: SDQ – Shoulder Disability Questionnaire; SPS – Shoulder Pain Score; SRQ – Shoulder Rating Questionnaire; PPR – Patient-perceived recovery; SF-36 – Short-form 36; IRCS – Individual Relative Constant Score.
